# Direct Selective Oxidative Functionalization of C–H Bonds with H_2_O_2_: Mn-Aminopyridine Complexes Challenge the Dominance of Non-Heme Fe Catalysts

**DOI:** 10.3390/molecules21111454

**Published:** 2016-10-31

**Authors:** Roman V. Ottenbacher, Evgenii P. Talsi, Konstantin P. Bryliakov

**Affiliations:** 1 Chemistry Department, Novosibirsk State University, Pirogova 2, Novosibirsk 630090, Russia; ottenbacher@catalysis.ru (R.V.O.); talsi@catalysis.ru (E.P.T.); 2 Boreskov Institute of Catalysis, Pr. Lavrentieva 5, Novosibirsk 630090, Russia

**Keywords:** C–H functionalization, enzyme models, hydrogen peroxide, manganese, mechanism, oxidation

## Abstract

Non-heme iron(II) complexes are widespread synthetic enzyme models, capable of conducting selective C–H oxidation with H_2_O_2_ in the presence of carboxylic acid additives. In the last years, structurally similar manganese(II) complexes have been shown to catalyze C–H oxidation with similarly high selectivity, and with much higher efficiency. In this mini-review, recent catalytic and mechanistic data on the selective C–H oxygenations with H_2_O_2_ in the presence of manganese complexes are overviewed. A distinctive feature of catalyst systems of the type Mn complex/H_2_O_2_/carboxylic is the existence of two alternative reaction pathways (as found for the oxidation of cumenes), one leading to the formation of alcohol, and the other to ester. The mechanisms of formation of the alcohol and the ester are briefly discussed.

## 1. Introduction

Given increasing demand for efficient and scalable methods of fine organic syntheses (mostly the syntheses of natural products, pharmaceuticals, biologically active molecules), the design of rigorous synthetic approaches, ensuring precise control of the carbon skeleton and oxidation state, continues to be one of the foremost challenges of synthetic chemistry. In the last years, selective catalytic oxidations of unactivated C–H bonds have attracted great attention: this approach, if implemented on preparative scale, could provide easy and efficient methodologies for the directed introduction of oxygen atom into complex organic molecules at late stages of multistep syntheses, on the basis of novel synthetic strategies [1,2,3,4,5,6,7,8,9,10,11]. Selective C–H oxidation reactions may lead to formation of new C–C and C–X (e.g., C–O, C–N, C–S, C–halogen, etc. [10]) bonds; in this paper, we will solely focus on the *oxygenation* processes, converting alkane groups into C–OH or C=O functionalities.

Metal-mediated *C–H activation* (including C–H bond cleavage and formation of C-metal *σ*-bonds) has been known for decades; one could mention in this context the works of Shilov [1,8,12], Periana [1,8,13], and others [1,8], dating back to the 1960s–1980s. Typically, C–H activation reactions require that the substrate should first coordinate to the catalytic complex of transition metal (Pt, Rh, Ir, etc.), which is relatively easy if the substrate has *π* electrons. In contrast to unsaturated substrates, alkanes have only *σ* electrons and thus are poor nucleophiles, coordinating only weakly to metals [8], which complicates the C–H activation step. On the other hand, to obtain a functionalized molecule, the activation step should be combined with subsequent functionalization step, which may represent some obstacles [8]. That is why in the last years, *direct* C–H oxofunctionalization, which does not imply the intermediate formation of a metal-carbon bond, has attracted close attention as a promising strategy for the selective oxidative modification of challenging substrates like alkanes. An additional advantage of the direct C–H oxidations is that most of them rely on catalysts based on complexes of cheap and abundant first-row transition metals (Fe, Cu, Mn, V, etc.).

Nowadays, the design of homogeneous catalysts for the direct C–H oxygenation largely follows the biomimetic approach, which implies modeling the catalytic functions of natural metalloenzymes (like cytochrome P450 or methane monooxygenase) with synthetic low-molecular weight complexes. It is not surprising that the major part of reported examples of catalytic C–H oxidations deal with catalysts based on complexes of Fe—the latter is the most widespread metal in natural metalloenzymes [14,15,16,17,18,19,20,21,22,23,24,25,26,27]. Most often, H_2_O_2_ is used as the “green” oxidant.

**Figure 1:**
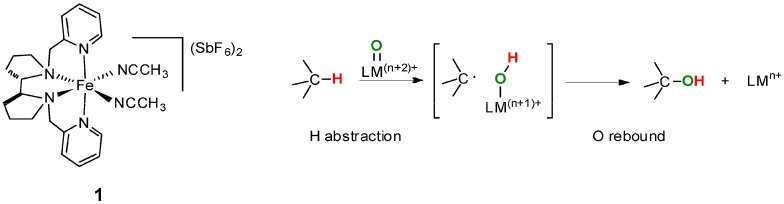
The structure of White’s catalyst **1** (**left**) and the rebound-mechanism (**right**).

Today, non-heme (essentially nonporphyrinic) Fe complexes dominate the area, due to the versatility of the ligand structures, and the rich possibilities of their structural modifications. First examples of non-heme-Fe-catalyzed oxidations appeared in early 1990s [24,28]; however, the breakthrough was achieved after 2007, when White with co-workers contributed a series of milestone works, presenting the bipyrrolidine-derived non-heme iron catalyst **1** (Figure 1) and its structural analogs, ensuring reasonably high level of predictability in the selective oxidation of C(sp^3^)-H groups [29,30,31,32,33,34]. In competitive contribution, Costas and co-workers showed that the introduction of additional steric crowd at the pyridine moieties, as well as manipulating with the symmetry of the chiral ligand can divert the oxidation selectivity from 3° C(sp^3^)-H bonds to stronger 2° C(sp^3^)-H bonds, which is critical for the selective oxygenation of complex molecules such as natural products [35,36,37,38]. The mechanism of non-heme iron catalyzed oxidations has been extensively studied experimentally [39]; it has now been accepted that the C–H oxidation proceeds via the classical rebound mechanism [40] (Figure 1), with participation of the elusive oxoperferryl species [41,42,43,44,45,46,47,48,49].

In contrast to non-heme Fe complexes, the catalytic activity (as well as the mechanism of catalytic action) of structurally related Mn complexes have so far been much less studied. Nevertheless, the Mn based catalyst systems for C–H oxidation, reported so far, demonstrate good practical promise; the Mn-catalyzed C–H oxidation mechanism seems to be mostly similar to that for the Fe-mediated direct C–H oxidation. In this contribution, we will briefly summarize the related synthetic and mechanistic data published to date, and discuss a new mechanistic feature, previously unobserved for Fe-catalyzed C–H oxidations, which seems to be characteristic of Mn-mediated C–H oxidations.

## 2. C–H Oxidations with H_2_O_2_ in the Presence of Mn Porphyrins

**Figure 2:**
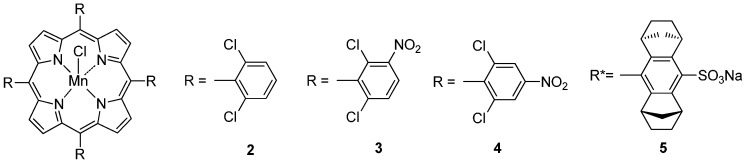
Structures of Mn porphyrins, studied in C–H oxidations.

In 1986, Mansuy with co-workers reported the catalytic oxidation of alkanes with H_2_O_2_ in the presence of Mn porphyrin complex **2** (Figure 2) [50]. Upon slow addition of H_2_O_2_ (5 equiv.) to the acetonitrile:dichloromethane solution of cyclohexane, catalyst **2** (2.5 mol %), and imidazole as additive (60 mol %), led to the formation of cyclohexanol and cyclohexanone (Table 1, entry 1) [50]. The 3:1 cyclohexanol:cyclohexanone ratio, the high (11.5) 3°:2° adamantane oxidation selectivity, the absence of effect of dioxygen on the oxidation outcome, and selective (non-statistical) oxygenation of heptane were indicative of a metal-mediated rather than radical-driven oxidation mechanism [51]. The authors hypothesized that the oxidation could be performed by the Mn^V^=O intermediate, the role of imidazole consisting in promoting the heterolytic cleavage of the O–O bond, to form the manganese-oxo species [52].

Banfi with co-workers considered a series of Mn-porphyrin complexes **2**–**4** as alkane oxidation catalysts and found that the introduction of a nitro group (**3** and **4** vs. **2**) resulted in an increase of activity and stability of the catalysts, eventually resulting in higher yields of oxygenated products [53]. It was shown that other heterocyclic bases may be efficiently used as additives, in combination with benzoic acid (Table 1, entries 2, 3). The proposed mechanism assumed the key role of a “Mn^V^=O” intermediate [53,54]. Complex **2** and its Br_8_-analog also demonstrated the capability of catalyzing the oxidation of aromatic compounds (such as anisole, naphthalene, phenantrene) with H_2_O_2_, in the presence of imidazole as additive (Table 1, entries 4, 5) [55]. The yields were low, and the high selectivities were only achieved at high substrate/oxidant/Mn ratios (typically 700:10:1). The hydroxylation of anisole occurred preferentially at the *p*-position; furthermore, some *O*-demethylation was detected (Table 1, entry 5) [55]. Mansuy with co-workers found that ammonium acetate is a more efficient co-catalyst than imidazole [56], the use of CH_3_COONH_4_ led (under similar conditions) to higher alkane conversions (but lower alcohol/ketone ratios, Table 1, entries 6, 7).

More recently, Simonneaux with co-workers developed the first Mn-porphyrin catalyzed enantioselective benzylic hydroxylations with H_2_O_2_ in the presence of catalyst **5** (Figure 2) [57]. The reactions could be readily performed in aqueous methanolic solutions, due to the good solubility of complex **5**. Enantioselectivities ranging from 32% to 57% *ee* were reported (Table 1, entry 8), and the catalyst performed up to 40 turnovers. The occurrence of asymmetric induction is evidence of non-radical hydroxylation mechanism. The highest enantioselectivities were documented for those substrates that ensured the highest alcohol selectivities at the same time (e.g., for the oxidation of 4-ethyltoluene: 97% conversion, alcohol/ketone = 93:7, enantioselectivity 57%), which rules out the possibility for generation of *ee* at the kinetic resolution step. SO_3_Na was the best substituent; its replacement with H, NMe_2_, or NO_2_ afforded less chemo- and enantioselective catalysts [58]. Conducting the reaction in a biphasic system (CH_2_Cl_2_/H_2_O_2_) deteriorated the oxidation outcome [58].

To date, the catalysts for C–H oxidation on the basis of Mn porphyrin complexes have been very rare (focusing on complex **2** or its modifications); the catalytic reactions have some characteristics of metal-mediated (non-radical) process (presumably driven by high-valent Mn=O species [21]). However, the mechanism of Mn-porphyrin-catalyzed C–H hydroxylations with H_2_O_2_ has not yet been reliably established.

## 3. C–H Oxidations with H_2_O_2_ in the Presence of Mn Complexes with Me_3_tacn Derived Ligands

**Figure 3:**
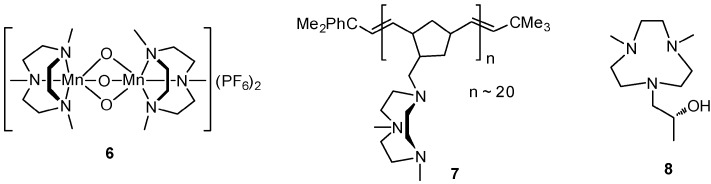
Structures of Mn complexes with Me_3_tacn derived ligands.

The selective C–H oxidations with H_2_O_2_ in the presence of non-porphyrinic Mn complexes have so far been extensively developed. The milestone in the area had been the communication of Shul’pin and Lindsay Smith [59,60] that dinuclear Mn(IV) complex **6** (Wieghardt’s complex [LMn^IV^(O)_3_Mn^IV^L](PF_6_)_2_, where L = 1,4,7-trimethyl-1,4,7-triazacyclononane, Me_3_tacn [61], Figure 3) catalyzed the oxidation of alkanes (hexane, cyclohexane, cycloheptane) with H_2_O_2_. In acetonitrile, catalyst **6** was very efficient, performing up to 1350 turnovers (Table 2). Carboxylic acid was reported to be an essential catalytic additive, and acetic acid was the best in the series [60]. The addition of radical scavenger BrCCl_3_ dramatically suppressed the yield of oxidized products. On the other hand, formation of sizable amounts of alkyl hydroperoxides was detected, and partial erosion of stereochemistry upon the oxidation of *cis*- and *trans*-1,2-dimethylcyclohexanes was documented, suggesting considerable impact of free radical driven oxidation pathways [62].

Catalyst **6** appeared to be extremely efficient in the oxidation of light alkanes (methane, ethane: Table 2, entry 2), as well as higher normal and branched alkanes at room temperature (performing up to 3100 turnovers) [63,64].

A polymer-bound complex was prepared starting from Me_3_tacn-derived ligand **7** (Figure 3) and Mn(OAc)_2_, which also conducted alkane oxygenation with H_2_O_2_/AcOH, demonstrating a lower activity as compared to its homogeneous prototype [65]. The reactivity profile of the polymer-bound catalyst was somewhat different from that of the dinuclear catalyst **6**, in particular higher *k*_H_/*k*_D_ (2.8 vs. 1.3; cyclohexane/cyclohexane-d_12_) and different selectivity for the hydroxylation of isooctane were documented [65].

Shul’pin with co-workers studied systematically the role of additives. Without additives, Mn complex **6** did not react with H_2_O_2_ in acetonitrile. However, even small amounts of acetic acid promoted both the catalytic decomposition of hydrogen peroxide and the alkane oxidation. If tiny amount of base was added to the acetic acid, the catalase activity of the **6** substantially increased, while the oxygenase activity decreased [66,67].

An interesting result was documented for the oxidation of alkanes with H_2_O_2_ in biphasic systems (H_2_O/alkane, without organic solvents) and in CH_3_CN, in the presence of **6** as catalyst, and oxalic acid as additive [68]. The latter, used as co-catalytic additive, led to higher hydroxylation stereospecificities than acetic acid (*cis*-1,2-dimethylcyclohexane oxidation, 80% retention of configuration, *RC*, with oxalic acid vs. 48% *RC* with acetic acid). Alkylhydroperoxides were the primary reaction products; they decomposed in the course of the reaction to the corresponding ketones and alcohols [68]. Crucially, screening a series of different (including aromatic) carboxylic acid additives revealed pyrazine-2,3-dicarboxylic and trifluoroacetic acids as extremely efficient co-catalysts [69,70].

Kim and co-workers studied the reactivity of the catalyst system **6**/H_2_O_2_ (with the Mn catalyst prepared in situ) towards cyclohexane in aqueous acetone in the presence of several carboxylic acids and their sodium salts [71]. In all cases, cyclohexanone was the major reaction product. Interestingly, in the presence of sodium oxalate, the oxidation of hexane demonstrated a high selectivity for hexanones (Table 2, entry 3), and the oxidation of *cis*- and *trans*-1,2-dimethylcyclohexanes was highly stereoretentive (>99% *RC*), which, in combination with the relatively high *k*_H_/*k*_D_ for cylcohexane oxidation (2.7), may be considered as evidence against radical type oxidation mechanism [71].

Srinivas and co-workers examined the benzylic oxidations in the presence of a Mn complex prepared in situ from Me_3_tacn and MnSO_4_ [72]. Ethylbenzene oxidations with H_2_O_2_ proceeded with good acetophenone selectivities, while the addition of carboxylate buffers increased the yield of 1-phenylethanol (Table 2, entry 4). High ketone selectivities were reported for benzylic oxidations, catalyzed by **6**, in the presence of trichloroacetic acid [73].

A set of Me_3_tacn derivatives, including optically pure structures of the type **8** (Figure 3), were prepared and tested as ligands in Mn-catalyzed oxidation of isopropyl alcohol and several alkanes with H_2_O_2_, either with oxalate or with ascorbic acid as co-catalysts [74].

Overall, C–H oxidations with H_2_O_2_ in the presence of Mn complexes, ligated by Me_3_tacn derivatives, has been rather extensively studied. To date, there are no decisive data on the common oxidation mechanism, which, owing to the involvement of atmospheric dioxygen, appears to be very complex. The provisional mechanistic picture can be found in [62,66]. In [65,66], one can also find a useful synopsis of early Mn-Me_3_tacn type catalyst systems, capable of conducting various oxidative transformations, using H_2_O_2_ as oxidant.

## 4. C–H Oxidations with H_2_O_2_ in the Presence of Mn Complexes with N,O Donor Ligands

**Figure 4:**
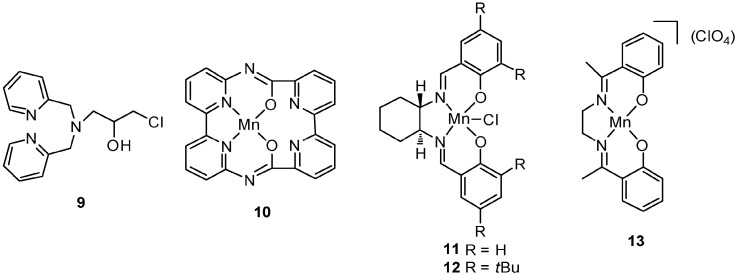
Structures of Mn complexes with N,O-donor ligands.

Fernandes and co-workers reported the water-soluble manganese(II) complex with ligand **9** (Figure 4), of the type [(**9**)Mn(*η*_1_-NO_3_)(*η*_2_-NO_3_)], which catalyzed the oxidation of cyclohexane with H_2_O_2_ (at 50 °C) in CH_3_CN or in *t*BuOH [75]. Moderate efficiencies were reported (up to 62 turnovers), with cyclohexyl hydroperoxide as the major product [75]. The cyclohexanol/cyclohexanone ratios were in the range 1.3-1.4, indicating a sizeable contribution of a radical oxidation pathway.

Ogawa and co-workers contributed Mn complex **10** (Figure 4) with a macrocyclic ligand, which catalyzed the oxidation of C–H groups in different hydrocarbons (cyclohexane, cyclooctane, ethylbenzene, benzene), performing up to 9 catalytic cycles without any co-catalytic additives [76]. For the oxidations of cyclohexane and cyclooctane, alcohol/ketone ratios of 19 and 8.2 were documented, without reported formation of cycloalkyl hydroperoxides [76].

In 1997, Mn-Salen type complexes **11** and **12** were mentioned to catalyze the oxidation of ethylbenzene (preferentially to acetophenone) with H_2_O_2_; it is questionable, however, whether this oxidation can be considered “catalytic” since the reported turnover numbers (TNs) were less than unity [77]. Much later, complex **13** was shown to catalyze benzylic oxidations with unexpectedly high efficiency (up to several thousand TN) in acetonitrile at room temperature, without any co-catalysts, affording ketones with high selectivity (Table 2, entry 5) [78]. The reaction required a high excess of H_2_O_2_ (10–20 mol per mol of substrate). Apparently, pronounced decomposition of (added in one portion) hydrogen peroxide occurred. A series of structurally related salen and salan (tetrahydrosalen) complexes were studied as catalysts in the oxidation of diphenylmethane [79]. The ligand structure has been shown to have crucial effect on the oxidation selectivity (the benzophenone:diphenylmethanol ratio varying from 100:0 to 15:85).

## 5. C–H Oxidations with H_2_O_2_ in the Presence of Mn Complexes with Aminopyridine Ligands

**Figure 5:**
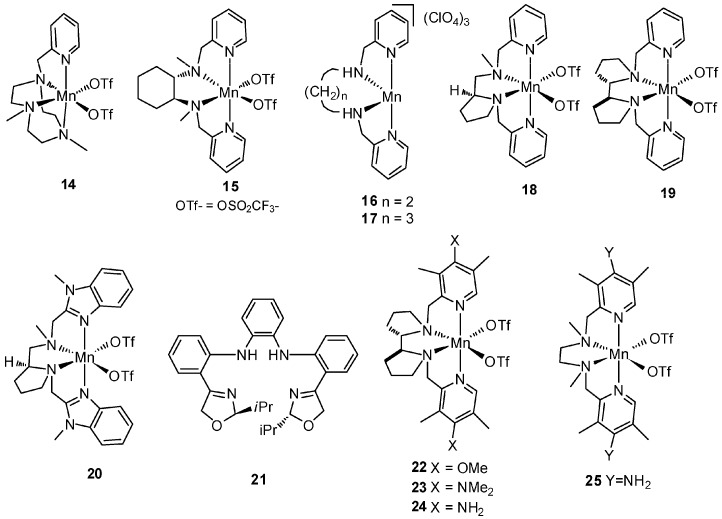
Structures of Mn complexes with aminopyridine ligands.

The current renascence of the Mn catalyzed selective C–H oxidations with H_2_O_2_ is associated with the appearance of Mn aminopyridine complexes. Initially, Costas and co-workers reported that complexes [(Me_2_Py-tacn)Mn(CF_3_SO_3_)_2_] (**14**) and [((*S,S*)-bpmcn)Mn(CF_3_SO_3_)_2_] (**15**) (Figure 5) demonstrated moderate oxidation reactivity towards *cis-*1,2-dimethylcyclohexane (with 1% and 8% yields, respectively) [80]. We also have to mention the report by Golchoubian and Ghasemi, who examined the oxidation of diphenylmethane with a series of Mn complexes with N_4_-donor ligands, including two aminopyridine, ligands (complexes **16**, **17**) [79]. With both complexes, alcohol was preferentially formed [79] (Table 3, entry 1).

Bryliakov and co-workers documented excellent catalytic efficiencies in the oxidations of a variety of 2° and 3° alkane C–H groups with H_2_O_2_, catalyzed by complexes **15**, **18** and **19** in the presence of acetic acid [81]. The Mn complexes performed up to 970 catalytic cycles. Cyclohexanol/cyclohexanone ratios of 4.9-5.1 and high 3°/2° selectivities of adamantane oxidation (40–49) pointed to a metal-mediated rather than a radical-driven oxidation mechanism. The high sensitivity of the oxygen-transferring species to electronic effects, and high selectivity and stereospecificity (>99% *RC*) in the oxidation of *cis*-1,2-dimethylcyclohexane also support the metal-mediated oxidation mechanism [81]. Under “practical” conditions, methylenic CH_2_ groups preferentially converted to ketone functionalities (Table 4, entry 1) [81].

More recently, Sun with co-workers reported the catalyst system based on complex **20** (Figure 5) [82]. In the presence of acetic acid as additive, the latter efficiently (up to 154 TN) catalyzed the oxygenation of secondary benzylic and aliphatic C–H bonds, with preferential formation of ketones (Table 3, entry 2). The same catalyst has shown good catalytic efficiency in the oxidation of secondary alcohols, performing up to 4700 catalytic turnovers [83]. Another laboratory has reported the oxidation of alcohols with H_2_O_2_ in the presence of Mn complex, formed in situ from ligand **21** (Figure 5) and Mn(OTf)_2_ [84], with substantially lower catalytic efficiencies. The authors screened a series of different carboxylic acid additives, of which adamantane carboxylic acid was chosen as ensuring the highest yield of ketone. Interestingly, the oxidation of substrates bearing both olefinic and primary or secondary alcoholic moieties, proceeded selectively towards the formation of *α*,*β*-unsaturated carbonyl compounds [84].

Bryliakov and co-workers synthesized complexes **22** and **23**, bearing electron-donating substituents, that exhibited superior efficiencies (as compared with catalysts **15**, **18**, **19**) in benzylic oxidations (Table 3, entries 6 and 7) [85]. Cumenes were oxidized preferentially into the corresponding alcohols, while ethylbenzene afforded mostly acetophenone. 

Recently, a comparative study by Malik and co-workers revealed the most evident advantages of the catalyst systems based on the [((*S*,*S*)-pdp)Mn(OTf)_2_] type complexes (**19**, **20**, **22**–**24**, Figure 4) [86]. Within the range of practically promising catalyst systems studied, the Mn-aminopyridine catalysts (1) are used in very small amount (0.1 mol %; structurally similar Fe complexes are typically used in up to 15 mol % loadings [29,30,31,32,33,34]); (2) require a small (1.3 equiv.) excess of the green oxidant—commercially available 30% aqueous H_2_O_2_; and (3) exhibit reasonably high yields in the oxidation of differently *p*-substituted cumenes, ensuring high cumyl alcohol selectivity [86].

**Scheme 1:**
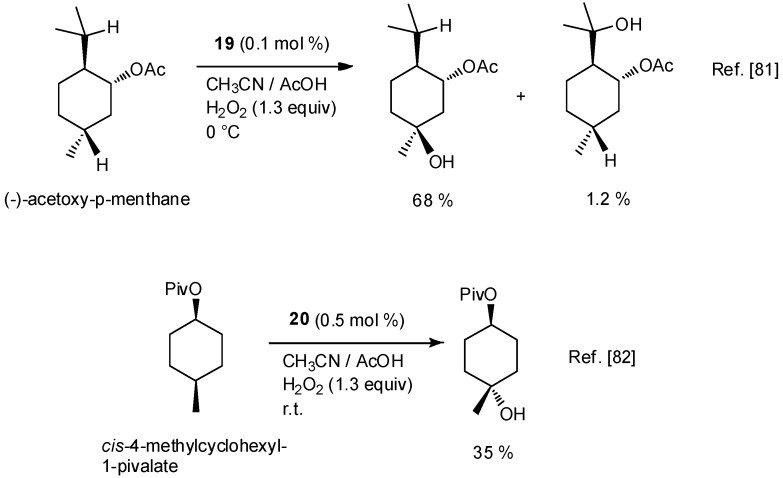

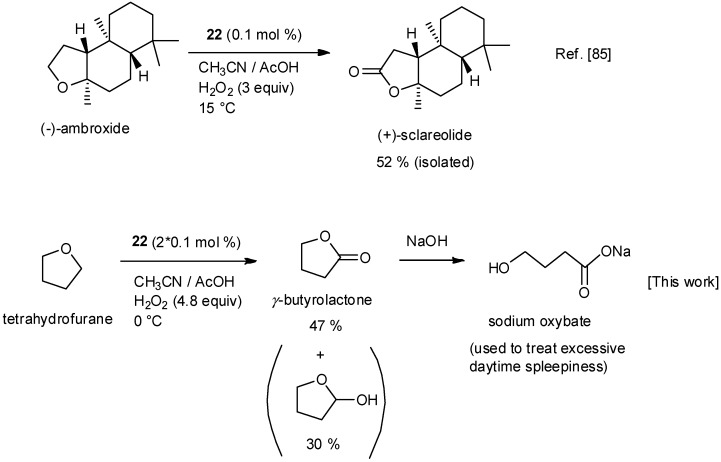
Examples of selective C–H oxidations of practical synthetic relevance in the presence of aminopyridine Mn complexes.

So far, Mn catalyzed selective oxidative syntheses of complex organic molecules, such as natural products, or biologically active compounds, have been rarely reported. A few procedures are collected in Scheme 1. We expect the emergence of other practically relevant examples in the near future.

## 6. Elucidation of the Mechanism of C–H Oxidations with H_2_O_2_ in the Presence of Mn Complexes

So far, experimental data on the mechanism(s) of C–H oxygenation with H_2_O_2_ in the presence of Mn complexes have been rather restricted in the literature. On the basis of indirect data, it has been generally assumed (see Section 2) that high-valent Mn-oxo complexes (presumably [(L)Mn^V^=O] or [(L^•+^)Mn^IV^=O]) are the most likely active species in the catalyst systems based on Mn porphyrins/H_2_O_2_/additive (for mechanistic considerations and precedents of porphyrinic Mn^V^O complexes see [21,39,52,57] and references therein.

On the contrary, catalyst systems based on Mn-Me_3_tacn type complexes exhibit various characteristics of radical-type mechanism (Section 3), which may be evidence of significant contribution of free-diffusing-radicals into the oxidation outcome.

**Scheme 2:**
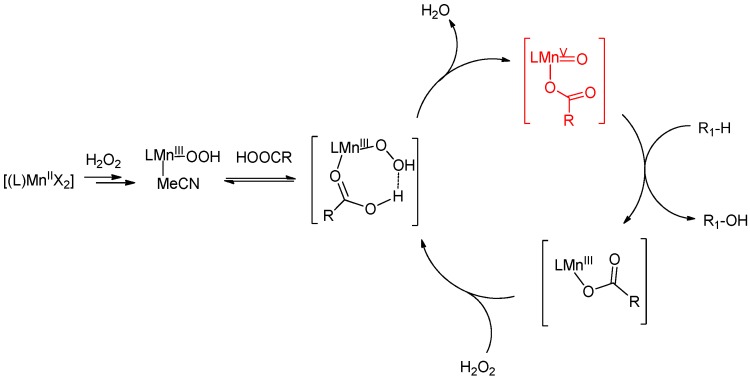
Proposed catalytic cycle for the selective C–H oxidations with H_2_O_2_ in the presence of aminopyridine manganese complexes and carboxylic acid RCOOH.

**Scheme 3:**
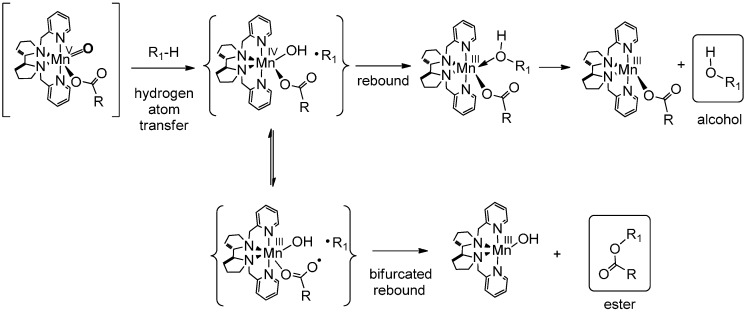
The proposed mechanism of Mn catalyzed C–H hydroxylation.

To date, it is only the mechanism of catalytic C–H hydroxylation on Mn-aminopyridine catalysts that has been studied in more detail [85]; in particular, the analysis of kinetic isotope effect (*k*_H_/*k*_D_), isotopic labeling data, and linear free energy relationships has been reported. The crucial findings were the observation of (1) a primary *k*_H_/*k*_D_ of 3.5–3.9 for the oxidation of cumene/*α*-^2^H-cumene, indicating that the hydrogen atom lies on the reaction coordinate and participates in C–H bond breaking step; (2) linear Hammett correlation for the oxidation of a series of *p*-substituted cumenes, with the *ρ*^+^ of −1.0, witnessing electrophilic active species, and electron-deficient transition state; (3) linear correlation between the C–H bond dissociation energies (BDEs) of the substrates and the logarithms of rates of their oxidations, reflecting a common oxidation mechanism; and (4) partial incorporation of ^18^O from added ^18^O-labeled water into the hydroxylated product, supporting the “water-assisted” [87,88] pathway for the formation of active, presumably oxomanganese(V), species [85]. In the presence of carboxylic acid additive, the mechanism of formation of the active species is diverted to the “carboxylic acid assisted” one (Scheme 2) [44,89,90,91,92]. The hydroxylation step has been concluded to proceed via the rebound mechanism (Scheme 3, top). This mechanistic landscape apparently dominates for the oxidation of unactivated C–H groups in aliphatic compounds.

Very recently, the mechanism of formation of the minor oxidation product—cumyl acetate has been studied in detail [93]. Counter to the earlier expectations [85], the possibility of esterification of the initially formed alcohol has been ruled out since separate experiments have shown that under the oxidation conditions, cumyl alcohol does not react with acetic acid. Moreover, the isotopic labeling data witness that both oxygen atoms of the acetate stem exclusively from the acetic acid molecule, which does not conform to the common Fischer esterification mechanism [93].

It has been concluded that the only feasible possibility remaining is the direct formation of the ester after the H abstraction step, via transfer of the acetoxy radical to the intermediate carbon-centered organic radical. Based on the catalytic, isotopic labeling, and density functional theory (DFT) study, the authors have proposed the “bifurcated rebound mechanism” (Scheme 3, bottom) which explains the observed acetate formation [93]. One of the two alternative rebound pathways, conducted by the intermediate [(PDP)Mn^IV^–OH(OC(O)R)] (Scheme 3) leads to the alcohol product, and the other, associated with the intermediate [(PDP)Mn^III^–OH(OC(O^•^)R)]) affords the ester. The competitive ^•^OC(O)R/^•^OH transfer has so far been unprecedented for bioinspired oxidations in the presence of non-heme Fe and related Mn complexes, for which effect the term “bifurcated rebound mechanism” has been proposed [93].

In the series of the Mn complexes studied, catalyst **24** (with the strongest electron-donor substituents) afforded the highest alcohol/acetate ratio (Table 3, entry 8 vs. entries 6, 7). The oxidations of cumene with H_2_O_2_ in the presence of a series of different aliphatic carboxylic acids were conducted (Table 4) [93]. The yield of the ester was lower (and the alcohol/ester ratio was higher) for carboxylic acids with tertiary *α*-carbon, as compared to those with primary or secondary *α*-carbon (cf. entries 4–8 vs. 1–3). The highest alcohol/ester ratio of 44.7 has been documented for the oxidation with 2-ethylbutyric acid (EBA, Table 4, entry 8). The lowest yield of ester for the acids with tertiary α-C may be due to additional stabilization of the [(PDP)Mn^III^–OH(OC(O^•^)R)] intermediate, owing to the existence of tautomeric, α-C-centered radical species, attenuating its reactivity. The use of branched (with tertiary *α*-carbon atom) carboxylic acids can be recommended for practical hydroxylations, in order to improve the alcohol formation selectivity.

## 7. Experimental Conditions

Oxidation of tetrahydrofuran (THF) to *γ*-butyrolactone was performed as follows: a solution of freshly distilled THF (40.5 μL, 500 μmol) and of manganese complex **22** (0.5 μmol) in CH_3_CN (0.40 mL) and AcOH (28.6 *μ*L, 500 *μ*mol) was thermostatted at 0 °C. 30% aqueous H_2_O_2_ (1200 *μ*mol, dissolved in CH_3_CN, total volume 200 *μ*L) was delivered to this mixture with a syringe pump over 60 min at 0 °C. Then, the second portion of manganese complex **22** (0.5 *μ*mol) was added, followed by the syringe pump addition (over 60 min) of the second portion of H_2_O_2_ (1200 *μ*mol, 200 *μ*L of solution in CH_3_CN). The mixture was stirred for additional 2 h at 0 °C. An internal standard (1,4-dioxane) was added and the mixture was subjected to gas chromatography–mass spectrometry (GC-MS) analysis.

## 8. Conclusions and Outlook

Synthetic complexes of Mn with polydentate ligands demonstrate the ability to act as biomimetic catalysts of direct C–H oxidation with H_2_O_2_, converting organic molecules into the oxygenated products (alcohols, ketones, and others). To date, the available library of catalytically active Mn complexes is rather wide, providing catalysts suited for C–H oxidations, ranging from the oxidation of benzylic C–H groups, through unactivated aliphatic C–H, to C–H groups of arenes. The oxidations typically proceed in a selective (and sometimes enantioselective [57,58]) fashion.

As a general trend, Mn based catalysts demonstrate higher (even much higher) oxidation efficiencies as compared to their Fe based structural analogs, with retention of similarly high selectivity. Moreover, in some cases Mn catalysts require a smaller excess of H_2_O_2_, down to only 1.3 equiv. relative to substrate.

The mechanisms of Mn-catalyzed C–H oxidations (generally taken, i.e., the mechanism of active species formation and the mechanisms of C–H activation and functionalization) have so far been rather underinvestigated. The data available to date give evidence for close similarities between the mechanistic landscapes of C–H oxidations in the presence of Fe- and Mn-aminopyridine complexes; providing indirect proof for the oxomanganese(V) active species. An important distinctive feature of Mn-aminopyridine based catalysts is the possibility of existence of two alternative reaction pathways, one leading to the formation of alcohol, and the other to the ester. The mechanism of the ester formation has been proposed, assuming competitive ^•^OC(O)R/^•^OH transfer to the alkyl radical formed at the H abstraction step. This mechanism, unprecedented for bioinspired oxidations in the presence of non-heme Fe and related Mn complexes, is proposed to be called “bifurcated rebound mechanism”.

Mn based catalysts for selective C–H oxidations with H_2_O_2_ provide novel opportunities for direct (without coordination of the substrate to the catalyst or formation of metal-carbon bond) functionalization of C–H bonds. Although at the current, laboratory stage of their development, the oxidations with H_2_O_2_ have not yet been reported to generate technically useful amounts of required oxygenated materials [94], the authors believe that Mn based “green” C–H oxidation systems will go beyond the laboratory scale in the near future.

## Figures and Tables

**Table 1:** Examples of C–H oxidations catalyzed by porphyrinic Mn complexes.

No.	Cat.	Substrate	Additive	Products (yield ^a^)	Ref.
1	**2**	cyclohexane	imidazole	cyclohexanol (12), cyclohexanone (4)	[50]
2	**3**	cyclohexane	*p*-*t*Bu-pyridine/benzoic acid	cyclohexanol (233 ^b^), cyclohexanone (47 ^b^)	[53]
3	**4**	cyclooctane	*p*-*t*Bu-pyridine/benzoic acid	cyclooctanol (565 ^b^), cyclooctanone (235 ^b^)	[53]
4	**2**	naphthalene	imidazole	1-naphthol (3.5), 2-naphthol (0.4)	[55]
5	**2**	anisole	imidazole	*p*-OH-anisole (4.7), *o*-OH-anisole (0.35), phenol (0.2)	[55]
6	**2**	cyclohexane	imidazole	cyclohexanol (12.4), cyclohexanone (1.6)	[56]
7	**2**	cyclohexane	CH_3_COONH_4_	cyclohexanol (20), cyclohexanone (5.2)	[56]
8	**5**	*p*-Et-toluene	imidazole	*p*-acetyltoluene (2.8), 1-(*p*-tolyl)ethanol (37.2, 57% *ee*)	[57]

**^a^** Yield of oxygenated products is given in mol/mol Mn; ^b^ In the original publication, alkane conversion was reported.

**Table 2:** Examples of C–H oxidations catalyzed by Mn complexes with Me_3_tacn derived and with *N*,*O*-donor ligands.

No.	Cat.	Substrate	Additive	Products (yield ^a^)	Ref.
1	**6**	hexane	AcOH	hexanones (775), hexanols (575)	[59]
2	**6**	ethane (20 bar)	AcOH	EtOOH (260), EtOH (120), MeCHO (20)	[64]
3	**6**	hexane	Na oxalate	2-hexanol (4.5), 3-hexanol (4.5), 2-hexanone (29), 3-hexanone (29)	[71]
4	**6**	ethylbenzene	oxalate buffer	1-phenylethanol (48.5), acetophenone (102)	[72]
6	**13**	ethylbenzene	none	1-phenylethanol (7), acetophenone (683)	[78]

**^a^** Yield of oxygenated products is given in mol/mol Mn.

**Table 3:** Examples of C–H oxidations catalyzed by Mn aminopyridine complexes.

No.	Cat.	Substrate	Additive	Products (yield ^a^)	Ref.
1	**17**	diphenylmethane	none	benzophenone (250), diphenylmethanol (530)	[79]
2	**19**	cyclohexane	AcOH	cyclohexanol (28), cyclohexanone (842)	[81]
3	**20**	cyclohexane	AcOH	cyclohexanone (122)	[82]
4	**20**	1-Ph-ethanol	AcOH	acetophenone (4700)	[83]
5	**21**/Mn(OTf)_2_	cyclohexanol	Ada-COOH	cyclohexanone (38)	[84]
6	**19**	cumene	AcOH	cumyl alcohol (209), cumyl acetate (139)	[93]
7	**23**	cumene	AcOH	cumyl alcohol (560), cumyl acetate (75), acetophenone (30)	[85]
8	**24**	cumene	AcOH	cumyl alcohol (492), cumyl acetate (61), acetophenone (110)	[93]

**^a^** Yield of oxygenated products is given in mol/mol Mn.

**Table 4:**
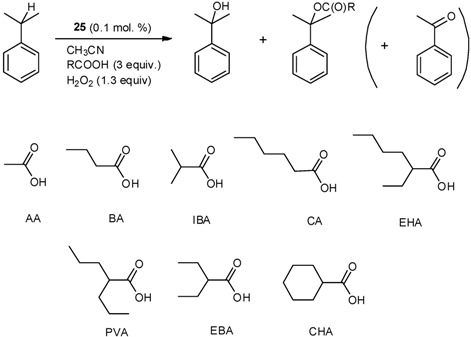
Oxidation of cumene with H_2_O_2_ in the presence of complex **25** and various carboxylic acids [93].

No	Additive	Conversion (%)	Yield of Alcohol/Acetate/Other ^a^ (%)	Alcohol/Ester Ratio
1	AA	82.9	62.9/12.1/7.9	5.2/1
2	BA	74.7	60.2/7.3/7.2	8.2/1
3	CA	56.5	46.6/4.3/5.6	10.8/1
4	CHA	34.3	30.3/2.0/2.0	15.4/1
5	IBA	61.8	51.7/3.2/6.9	16.2/1
6	EHA	29.8	26.1/1.6/2.1	16.3/1
7	PVA	38.6	34.5/0.9/3.2	36.7/1
8	EBA	39.2	35.4/0.8/3.0	44.7/1

**^a^** side product: acetophenone.
